# WHY DO OLDER WORKERS WITH CHRONIC HEALTH CONDITIONS PREFER TO RETIRE EARLY?

**DOI:** 10.1093/geroni/igz038.165

**Published:** 2019-11-08

**Authors:** Anushiya Vanajan, Ute Bültmann, Kène Henkens

**Affiliations:** 1 Netherlands Interdisciplinary Demographic Institute, South-Holland, Netherlands; 2 University Medical Center Groningen, Groningen, Netherlands

## Abstract

Older workers experiencing chronic health conditions (CHCs) are more likely to retire early. Current literature, however, lacks knowledge on the different pathways through which CHCs stimulate retirement preference. Earlier research is highly fragmented. Some studies have found CHCs to impact vitality, work limitations, or subjective life expectancy. Others have found vitality, work limitations, or subjective life expectancy to predict retirement preferences. We present a comprehensive model in which we hypothesize that the effects of four CHCs - arthritis, cardiovascular disease, sleep disorders, and psychological disorders - on retirement preferences are differentially mediated by vitality, health-related work limitations, and subjective life expectancy. We analyzed data from 6,294 older workers (60 – 65 years) in the Netherlands. Effects of CHCs on older workers’ retirement preferences were mediated by vitality, health-related work limitations, and subjective life expectancy. The main mediation pathway differed for each CHC. Severe health-related work limitations among older workers with arthritis (65.6% mediated) and cardiovascular disease (44.0%) predominantly guided their retirement preferences. Lower vitality levels mainly mediated retirement preferences of older workers with sleep (59.1%) and psychological disorders (52.9%). Lower subjective life expectancy was a significant mediation pathway (13.7%) for older workers with cardiovascular diseases. Extending working lives is a key public health and policy challenge. We show that health-related work limitations and vitality play a major role in determining retirement preferences of older workers experiencing CHCs. Since both mediators are modifiable, targeted interventions may not only extend the working lives of older workers, but also improve its quality.

